# Effects of particulate air pollution on tuberculosis development in seven major cities of Korea from 2010 to 2016: methodological considerations involving long-term exposure and time lag

**DOI:** 10.4178/epih.e2020012

**Published:** 2020-03-12

**Authors:** Honghyok Kim, Sarah Yu, Hongjo Choi

**Affiliations:** 1 BK21Plus Program in Embodiment: Health-Society Interaction, Department of Public Health Sciences, Graduate School, Korea University, Seoul, Korea; 2 School of Forestry and Environmental Studies, Yale University, New Haven, CT, USA; 3 Research Center, the Korean Institute of Tuberculosis, Korean National Tuberculosis Association, Seoul, Korea; 4 School of Health Policy and Management, Korea University College of Health Science, Seoul, Korea; 5 Department of Preventive Medicine, Konyang University College of Medicine, Daejeon, Korea

**Keywords:** Particulate matter, Tuberculosis, Air pollution, Time

## Abstract

**OBJECTIVES:**

Epidemiological evidence of associations between ambient particulate matter (PM) and tuberculosis (TB) risk is accumulating. Two previous studies in Korea found associations between air pollution—especially sulfur dioxide (SO_2_)—and TB. In this study, we conducted an annual time-series cross-sectional study to assess the effect of PM with an aerodynamic diameter less than 10 μm (PM_10_) on TB risk in seven major cities of Korea from 2010 to 2016, taking into account time lag and long-term cumulative exposure.

**METHODS:**

Age-standardized TB notification rates were derived using the Korea National TB Surveillance System. Annual average PM_10_ concentrations were obtained from annual Korean air quality reports. We applied a generalized linear mixed model with unconstrained distributed lags of exposure to PM_10_. We adjusted for potential confounders such as age, health behaviors, and area-level characteristics.

**RESULTS:**

Both average annual PM_10_ concentrations and age-standardized TB notification rates decreased over time. The association between cumulative exposure to PM_10_ and TB incidence became stronger as a longer exposure duration was considered. An increase of one standard deviation (5.63 μg/m^3^) in PM_10_ exposure for six years was associated with a 1.20 (95% confidence interval, 1.17 to 1.22) times higher TB notification rate. The marginal association of exposure duration with the TB notification rate was highest at four and five years prior to TB notification. This association remained consistent even after adjusting it for exposure to SO_2_.

**CONCLUSIONS:**

The findings of this study suggest that cumulative exposure to PM_10_ may affect TB risk, with a potential lag effect.

## INTRODUCTION

Although a relationship between indoor air pollution and tuberculosis (TB) has been established, the impact of outdoor air pollution on TB development has been insufficiently examined [1-3]. Epidemiological evidence of associations between ambient particulate matter (PM) and TB risk is accumulating [4-7]. Outdoor air pollution originating with traffic and industrial emissions may affect the susceptibility of the lung to infections by altering immunological processes such as the function of pulmonary macrophages, polymorphonuclear leukocytes, and epithelial cells [8]. However, two epidemiological studies conducted in Korea failed to find associations between ambient air pollutant concentrations and TB incidence, with the exception of sulfur dioxide (SO_2_) [9,10]. While the biological mechanisms described above suggest that there may be a time lag between PM exposure and TB incidence, time lag has been understudied.

The aim of this study was to obtain a better understanding of how outdoor air pollution affects TB risk, by evaluating the time lag and association between long-term exposure to ambient PM with an aerodynamic diameter less than 10 µm (PM_10_) and TB incidence in seven major cities of Korea (Seoul, Busan, Daegu, Incheon, Gwangju, Daejeon, and Ulsan) from 2010 to 2016.

## MATERIALS AND METHODS

Notified TB cases in the seven major cities were obtained from the Korean National Tuberculosis Surveillance System (KNTSS) for the period 2010-2016. In the World Health Organization’s global TB report, notified TB cases were used to estimate the incidence of TB in Korea by multiplying by a correction factor [11]. A previous study found that the completeness of TB notification was over 90% from 2012 to 2014 [12].

City-specific annual averages of PM_10_ and SO_2_ concentrations were obtained from the annual Korean air quality reports from 2006 to 2017 published by the Ministry of Environment in Korea. The annual levels were calculated from hourly measurements taken at government-managed monitoring stations in each city. To adjust for potential confounders, we used the number of hospital beds per 1,000 persons from 2010 to 2016, obtained from the Korean Statistical Information Service (KOSIS, https://kosis.kr) as a proxy measure of medical resources [13]. Statistics on the prevalence of smoking, prevalence of drinking, prevalence of physiciandiagnosed diabetes in adults over 30 years old, and population density were also obtained from the KOSIS. To adjust for differences in socioeconomic conditions across the seven cities, a deprivation index was used. Details for calculating the deprivation index have been described elsewhere [14]. We used Korea Census 2005 data from the KOSIS as a reference for calculating annual age-standardized TB notification rates.

We used an annual time-series cross-sectional design [15]. Our data resemble panel data, as the unit of study subjects was the city. Since the source population of TB cases in the KNTSS is the general population of the seven major cities of Korea, this study design may be seen as an ordinary time-series design in air pollution epidemiology with spatial variability of the exposure and outcome.

A generalized linear mixed model was applied to analyze the association between PM_10_ concentrations and the TB notification rate. A Poisson distribution was assumed for notified TB cases and a random intercept by city was considered. An unconstrained distributed lag model [16] with a lag period up to 5 years (i.e., *L*= 5) was applied to flexibly estimate the association between exposure to long-term PM_10_ and the TB notification rate as follows:

$$\log\left(E\left[Y_{t}^{i}\right]/P_{t}^{i}\right)\;=\;\beta_{0}\;+\;\gamma_{i}\;+\sum_{l=0}^{L}\beta_{l}X_{t-l}^{i}\;+\;\delta C_{t}^{i}$$

$Y_{t}^{i}$ is the number of notified TB cases by 10-year age groups (i.e., 0-19, 20-29, 30-39, 40-49, 50-59, 60-69, 70+) in city *i* for year *t*.$P_{t}^{i}$ is the population of city *i* in year *t*, which was included as an offset function. *β*_0_ + *γ_i_* is a random intercept for city *i*. $X_{t-l}^{i}$ represents PM_10_ concentrations in city *i* in year *_t-l_*, while *β_l_* represents the log-relative rate (RR) of *X_t-l_*. $C_{t}^{i}$ and δ are a matrix of potential confounders in city *i* for year *t* and their coefficients, respectively. We added some potential confounders to the model, including the distribution of age groups, the number of hospital beds per 1,000 persons, prevalence of smoking, prevalence of drinking, prevalence of diabetes mellitus, population density, and the deprivation index [17]. Age groups were included using dummy variables. The values of the other potential confounders were scaled using the z-score standardization method to estimate the model parameters. The estimated *β_l_* values in the model were transformed to indicate the association between PM_10_ and TB notification rate per an increase of one standard deviation (SD) of PM_10_ (5.63 µg/m^3^).

We conducted multiple sensitivity analyses. First, we adjusted the association between PM_10_ and the TB notification rate for SO_2_ concentrations because the aforementioned two Korean studies found an association between SO_2_ concentrations and TB incidence. However, since SO_2_ concentrations may indicate not only SO_2_ exposure, but also exposure to finer forms of PM [18,19], we compared how coefficients of PM_10_ changed after this adjustment was made and did not consider it as the main model. Second, we conducted the same analysis limited to TB cases aged 40 years or older in order to test the impact of exposure misclassification, because we did not have information on changes of residence in TB cases. Since younger people are more likely to change their residence for educational or occupational purposes, the subset analysis would yield higher estimates if non-differential exposure misclassification was more common in younger age groups.

We used the statistical software Microsoft R Open 3.5.3 (https://mran.microsoft.com/download) for the statistical analysis. The *lme4* package was used for the generalized linear mixed model.

### Ethics statement

This study was exempted from full review by the Institutional Review Board of the Korea National Tuberculosis Association (2019-KNTA-IRB-01) because it analyzed de-identified and encrypted data.

## RESULTS

**Figure 1.**
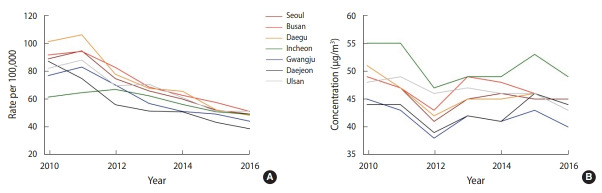
Time trends in the age-standardized tuberculosis (A) notification rate and (B) particulate matter with an aerodynamic diameter less than 10 μm levels in seven major Korean cities from 2010 to 2016.

A total of 120,280 notified TB cases were identified in the seven major Korean cities from 2010 to 2016. The age-standardized TB notification rates decreased over time in all seven cities (Figure 1A). The annual average PM_10_ concentrations also decreased over time in all seven cities (Figure 1B). The mean PM_10_ concentration from 2010 to 2016 ranged from 41.71 µg/m^3^ in Gwangju to 51.00 µg/m^3^ in Incheon.

**Figure 2.**
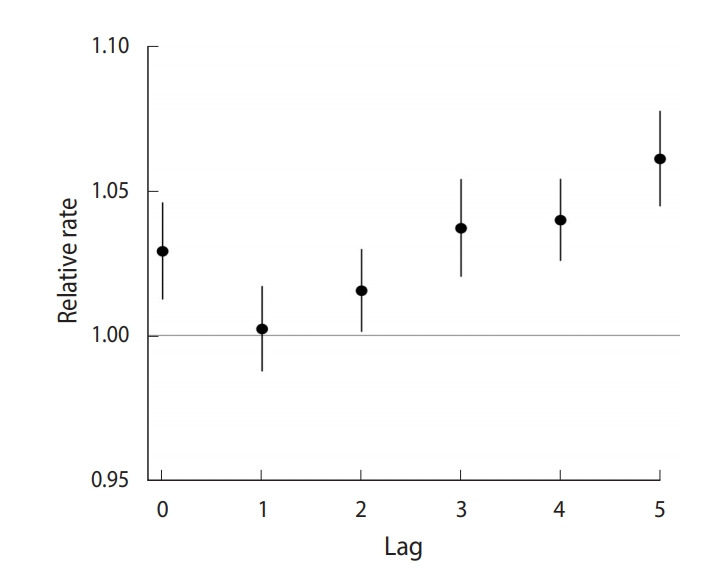
Associations of one standard deviation (5.63 μg/m^3^ ) increases in lagged particulate matter with an aerodynamic diameter less than 10 μm with the tuberculosis notification rate in seven major cities of Korea from 2010 to 2016.

Figure 2 presents the association between exposure to PM_10_ and the TB notification rate over various lag times. An increase of one SD (5.63 µg/m^3^) in the annual average PM_10_ concentration was associated with a 1.03 (95% CI, 1.01 to 1.05) times higher TB notification rate in the same year (lag 0). The marginal association between exposure to PM_10_ and TB notification rate was the highest for PM_10_ concentration five years prior to the TB notification rate (lag 5), with an RR of 1.06 (95% CI, 1.04 to 1.08).

Table 1 displays the cumulative associations between exposure to PM_10_ and the TB notification rate by different exposure durations. A longer exposure duration was associated with a higher cumulative association. A 5.63 µg/m^3^ increase of PM_10_ over six years was associated with the TB notification rate that was 1.20 (95% CI, 1.17 to 1.22) times higher.

When adjustment for unconstrained lags of SO_2_ was made, the estimated associations between exposure to PM_10_ and the TB notification rate slightly decreased. The highest marginal association (RR) was 1.04 (95% CI, 1.02 to 1.05) at lag 3, followed by 1.03 (95% CI, 1.01 to 1.05) at lag 0. Six years of PM_10_ exposure was associated with an RR of 1.17 (95% CI, 1.13 to 1.20) (Table 1). The analysis limited to TB cases aged 40 years or older found consistent estimated associations (data not shown).

## DISCUSSION

This study underscores the methodological importance of investigating the impact of outdoor air pollution on TB with regard to time lag and exposure duration. Two previous Korean studies failed to find associations between particulate air pollution and TB notification rates [9,10]. However, our study presented clear associations when cumulative exposure and the time lag between exposure and the active disease were considered.

We used TB notification data from the KNTSS as a proxy of TB incidence. More than 90% of diagnosed cases of TB from 2012 to 2014 were reported to the KNTSS, and almost 98% of them were reported within one year [12]. In light of this, our findings suggest associations between long-term exposure to PM and TB incidence in the seven major cities of Korea.

Exposure to outdoor air pollution may alter the lung environment and immune response by reducing levels of tumor necrosis factor-alpha and generating interferon-gamma [9]. Moreover, a study reported that diesel exhaust particles could downregulate the host gene expression induced by *Mycobacterium tuberculosis* (MTB) [20]. Alterations of the lung immune system may create biologically favorable conditions that lead to the reactivation of MTB [21]. For this reason, it is more plausible to consider the effects of long-term exposure to air pollutants on the deterioration of the lung immune response when investigating associations between particulate air pollution and active TB in the human body. Several studies have also considered long-term exposure and the lag between exposure and active TB. A United States study considered a 2-year lag, but identified no association between particulate pollution and active TB [22]. A study conducted in Taiwan considered a 1-year lag and found that particulate pollution was associated with TB [23]. Few studies, however, have considered risk differences over different time lags and exposure durations.

We found that the estimated associations between exposure to PM_10_ and the TB notification rate after adjusting them for SO_2_. However, this finding may not indicate confounding by SO_2_, but rather a slight over-adjustment, in that several air pollution epidemiologic studies in Korea have suggested that SO_2_ may act as a surrogate of exposure to finer forms of PM or PM emitted from combustion sources [18,19]. In light of this, the association between SO_2_ concentrations and TB incidence reported in the previous two Korean studies may support the association between exposure to PM and TB incidence.

Some limitations of this study deserve to be noted. First, individual-level confounding cannot be ruled out in our ecological study design, although we considered potential time-varying confounders in this study. Our findings should therefore be interpreted with caution. Nevertheless, our study may provide insights on the relationship between long-term exposure to PM and TB incidence in Korea, since it utilized TB notification data from the KNTSS, of which the source population is the general population of seven major cities of Korea. For future research, individual-level data analysis should be conducted to complement current ecological findings. Second, we could not use personal exposure measurements. Instead, we used representative city-level concentrations as an exposure metric. This type of exposure misclassification might have affected estimates toward the null in our study design [19,24,25]. Third, we did not have information on changes of residence in TB cases. The bias from exposure misclassification may be larger when a longer exposure duration is considered. This misclassification may be non-differential because people are likely to change their residence for economic reasons (e.g., occupation, financial issues). We examined whether the estimates changed when our analysis was restricted to only those aged 40 years or older (who were less likely to move than younger populations), and found consistent results, suggesting that bias from exposure misclassification may not have been severe. Fourth, we were not able to identify more precisely (e.g., within a year) when TB incidence actually occurred. Since TB cases may be notified to the KNTSS after hospital visits for TB, the use of hospital visits for TB in future research may enable a better understanding of the association between TB incidence and air pollution. Fifth, we adjusted our models for age, but age was considered in 10-year intervals. We could not rule out residual confounding by age within 10-year intervals.

The major lesson of this study is that the time lag between exposure and TB incidence and exposure duration should be considered when investigating the association between exposure to PM and TB incidence. We suggest that future epidemiological studies should consider the time interval between pollutant exposure and disease progression to obtain a better understanding of the relationship between outdoor air pollution and TB risk.

## Figures and Tables

**Table 1.** Cumulative associations between an increase of one SD (5.63 μg/m^3^) of exposure to PM_10_ and the TB notification rate by different exposure durations in seven major Korean cities from 2010 to 2016

Exposure duration (yr)	Cumulative RR (95% CI)^1^
PM_10_	
1	1.03 (1.01, 1.05)
2	1.03 (1.01, 1.05)
3	1.05 (1.02, 1.07)
4	1.09 (1.07, 1.11)
5	1.13 (1.11, 1.15)
6	1.20 (1.17, 1.22)
SO_2_-adjusted	
1	1.04 (1.01, 1.07)
2	1.01 (0.98, 104)
3	1.03 (0.10, 1.06)
4	1.12 (1.08, 1.16)
5	1.14 (1.10, 1.17)
6	1.17 (1.13, 1.20)

SD, standard deviation; PM_10_, particulate matter with an aerodynamic diameter less than 10 μm; TB, tuberculosis; RR, relative rate; CI, confidence interval.

1 The cumulative RR is exp(Σ*^L^_l_* = _0_ *β_l_*) for a 5.63 μg/m^3^ increase in PM_10_.
